# Evolution of Acridines and Xanthenes as a Core Structure for the Development of Antileishmanial Agents

**DOI:** 10.3390/ph15020148

**Published:** 2022-01-26

**Authors:** Carlos F. M. Silva, Diana C. G. A. Pinto, Pedro A. Fernandes, Artur M. S. Silva

**Affiliations:** 1 LAQV-REQUIMTE & Department of Chemistry, University of Aveiro, 3810-193 Aveiro, Portugal; silva.c@ua.pt (C.F.M.S.); artur.silva@ua.pt (A.M.S.S.); 2 UCIBIO, REQUIMTE, Departamento de Química e Bioquímica, Faculdade de Ciências, Universidade do Porto, 4169-007 Porto, Portugal; pafernan@fc.up.pt

**Keywords:** leishmania, tricyclic heterocycles, acridines, xanthenes

## Abstract

Nowadays, leishmaniasis constitutes a public health issue in more than 88 countries, affecting mainly people from the tropics, subtropics, and the Mediterranean area. Every year, the prevalence of this infectious disease increases, with the appearance of 1.5–2 million new cases of cutaneous leishmaniasis and 500,000 cases of visceral leishmaniasis, endangering approximately 350 million people worldwide. Therefore, the absence of a vaccine or effective treatment makes the discovery and development of new antileishmanial therapies one of the focuses for the scientific community that, in association with WHO, hopes to eradicate this disease shortly. This paper is intended to highlight the relevance of nitrogen- and oxygen-containing tricyclic heterocycles, particularly acridine and xanthene derivatives, for the development of treatments against leishmaniasis. Thus, in this review, a thorough compilation of the most promising antileishmanial acridine and xanthene derivatives is performed from both natural and synthetic origins. Additionally, some structure–activity relationship studies are also depicted and discussed to provide insight into the optimal structural features responsible for these compounds’ antileishmanial activity.

## 1. Introduction

Leishmaniasis is amongst the most neglected diseases in modern times, affecting mainly people from developing countries of the tropics, subtropics, and the Mediterranean basin. Approximately 350 million people are considered at risk of developing this disease [1]. This disease constitutes a public health issue in more than 88 countries, with an estimated world prevalence of 12 million cases covering all forms of leishmaniasis [2]. Moreover, these numbers tend to increase, since 1.5–2 million new cases of cutaneous leishmaniasis and 500,000 cases of visceral leishmaniasis appear each year, with a mortality rate of approximately 50,000 deaths annually. Epidemiologically, leishmaniasis consists of a complex vector-borne parasitic disease caused by roughly 20 *Leishmania* species and may be transmitted by more than 30 species of phlebotominae sand flies, an invertebrate vector [3,4]. From a clinical point of view, this infectious disease may be expressed in three major clinical forms, namely visceral leishmaniasis (VL) or kala-azar, cutaneous leishmaniasis (CL), and mucocutaneous leishmaniasis (MCL), the cutaneous and visceral forms being the most prevalent [5].

**Figure 1:**
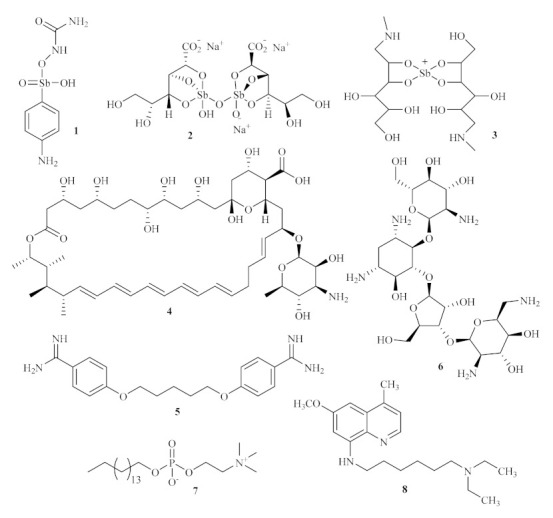
Currently used antileishmanial agents.

Since there is no effective vaccine, the treatment of leishmaniasis is solely dependent on chemotherapy, with organoantimonial compounds remaining the first line of treatment for all the forms of leishmaniasis for more than 60 years [6]. The first effective drug to treat leishmaniasis was urea stibamine **1** (Figure 1), discovered in 1912 but only described as effective against *L. donovani* in 1922 [7]. This massive breakthrough would then lead to the development and refinement of pentavalent antimonials [Sb(V)], such as the generic sodium stibogluconate **2**, also known as pentosam, or the branded meglumine antimoniate **3**, also known as glucantim, progressively reducing the side effects of the treatments against leishmaniasis. The need for safer and more effective medicines to treat leishmaniasis drove the search for new compounds, with amphotericin B (AmB) **4** (Figure 1) emerging as the first alternative to the usual pentavalent antimonials [1,8]. This medicine was initially extracted from *Streptomyces nodous*, a filamentous bacterium, in 1955, alongside amphotericin A, both isolated from a soil culture [9]. Furthermore, other antileishmanial agents, such as pentamidine **5** [10], paromomycin **6** [11], miltefosine **7** [12], or sitamaquine **8** [13], have also emerged as pharmaceutical alternatives for the treatment of leishmaniasis. However, the use of these aforementioned antileishmanial drugs has faced several limitations throughout the years, such as the emergence of several side effects or the increased incidence of resistance mechanisms of the parasites. The side effects include fever, chills, nausea, abdominal pain, or, more infrequently, cardiac and renal toxicity [14].

Based on the absence of appropriate treatment and an increase in resistance against the currently used medicines, the search for novel antileishmanial is starting to be in the spotlight inside the scientific community. Throughout the years, numerous scaffolds have been used as the core structure for the development of promising antileishmanial agents, with a long way to go until the discovery of an effective treatment against this disease [15,16,17]. Thus, this paper intends to compile and emphasize the potential of two major classes of compounds, namely acridines and xanthenes, to develop novel treatments against leishmaniasis, but also highlight a neglected disease and encourage the scientific community to devote more efforts to finding efficient treatment.

## 2. Acridines

**Figure 2:**
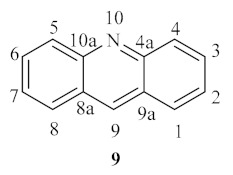
Acridine basic scaffold.

The class of acridine derivatives (Figure 2) constitutes an interesting group of nitrogen-containing tricyclic compounds that caught the scientific community’s attention, mainly due to its wide range of pharmaceutical properties [18,19]. This type of compound’s unique physical and chemical properties allows several derivatives to have been associated with numerous bioactivities, such as anti-inflammatory, anti-cancer, antimicrobial, antitubercular, antiparasitic, antimalarial, antiviral, and fungicidal activities. Furthermore, several methodologies have been developed to obtain a more extensive range of acridine-based compounds throughout the years. These methodologies include the Bernthsen synthesis, the first synthetic method for the obtention of an acridine, the Ullmann reaction, and the Friedländer synthesis [20,21]. Acridine derivatives can also be obtained by the manipulation of acylated diphenylamines, reduction of acridones, or the functionalization of 9-chloroacridines [20].

**Figure 3:**
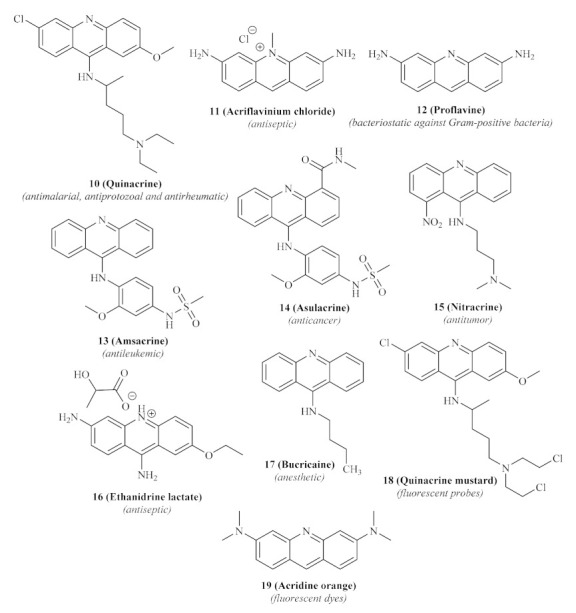
Acridine-based pharmaceutical agents and dyes.

Until today, numerous acridine derivatives have been used as pharmaceutical agents, such as quinacrine **10**, acriflavinium chloride **11**, proflavine **12**, amsacrine **13**, asulacrine **14**, nitracrine **15**, ethacridine lactate **16**, and bucricaine **17** (Figure 3). Additionally, this family of compounds has been widely used as fluorescent dyes, such as acridine orange **19**, as well as fluorescent probes, such as quinacrine mustard **18** [20].

Despite using acridine derivatives for pharmaceutical purposes, their potential use as antileishmanial agents remains unclear. However, next, we intend to demonstrate that there are already some reports of acridine derivatives with interesting antileishmanial effects. Thus, this might serve as proof that the acridine scaffold may be used for the development of novel acridine-based antileishmanial drugs and incentivize more studies involving this scaffold.

**Figure 4:**
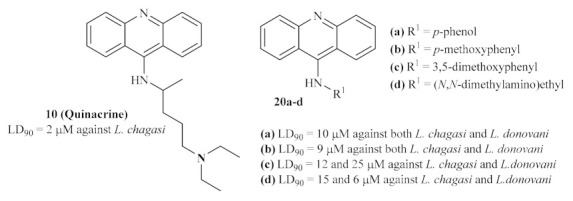
9-Aminated acridines active against leishmanial DNA topoisomerase II.

In the last decade of the 20th century, the acridine scaffold emerged as an exciting structural core for developing novel antileishmanial agents. Focusing their efforts on the leishmanial DNA topoisomerase II (TPII) as a promising molecular target, Werbovetz et al. assessed the efficiency of quinacrine **10** and several other 9-aminoacridines **20** (Figure 4) against DNA TPII of two *Leishmania* species, *L. donovani* and *L. chagasi* [22]. DNA topoisomerases are a widely distributed family of enzymes crucial for many essential biological processes, including DNA replication, transcription, recombination, and repair. Throughout the years, these enzymes have been indicated as chemotherapeutic targets for developing antibacterial and antiparasitic treatments, suggesting that the inhibition of these enzymes might consequently promote antileishmanial properties [23].

Their results demonstrated that several 9-substituted acridine derivatives, namely *p*-phenol **20a**, *p*-methoxyphenyl **20b**, 3,5-dimethoxyphenyl **20c**, (*N*,*N*-dimethylamino)ethyl 9-aminoacridine **20d**, and also the well-known antileishmanial agent quinacrine **10**, presented high levels of antileishmanial activity against the promastigote stage of the parasite, with lethal dose capable of killing 90% of parasite (LD_90_) values below 25 µM. Interestingly, except for *p*-phenol 9-aminoacridine **20a**, all of these active antileishmanial acridine derivatives also showed weak to no mammalian TPII inhibition, suggesting a selective mechanism of action solely against the enzyme of the *Leishmania* promastigotes. However, in our opinion, the authors must corroborate this hypothesis with an assay that shows these compounds’ effect on leishmanial TPII.

**Figure 5:**
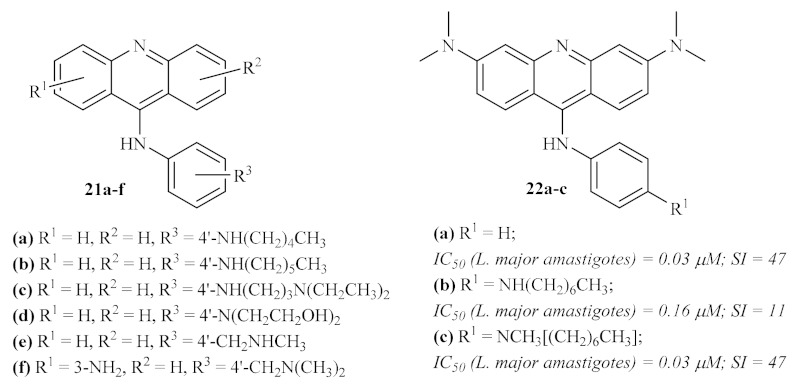
Most promising 9-anilinoacridines active against intracellular amastigotes of *L. major*.

Following the promising results of the previously mentioned 9-aminated acridines active against leishmanial DNA topoisomerase II, a series of 9-anilinoacridines was screened against macrophage-infected *L. major* [24]. By comparing the effect of some of these compounds on promastigotes and intracellular parasites, it becomes clear that derivatives active against the free form of the parasite are also able to act against the parasites inside the macrophages, being even more active against the intracellular microorganisms. Their results demonstrated that several 9-anilinoacridines **21** (Figure 5) show high levels of antileishmanial activity, killing more than 80% of intracellular parasites at 1 µM while maintaining moderate to low levels of mammalian toxicity. From this series of 9-anilinoacridines, the 4′-NHhexyl derivative **21.b** has been identified as the most promising antileishmanial compound, being one of the most active compounds while having the lowest level of toxicity. Interestingly, some structural features can be associated with both high levels of antileishmanial activity and low levels of toxicity, such as its lipophilicity or the presence of specific substituents, such as 3′-methoxy groups, with more promising values of toxicity.

In the final years of the 20th century, Gamage et al. continued their previous work with 9-anilinoacridines by synthesizing and evaluating against intracellular *L. major* amastigotes several other 4′-substituted 9-anilinoacridines **22** [25]. This study’s results corroborated some of the evidence already stated by this research group, such as the importance of a lipophilic electron-donating group at 4′-position of this type of derivatives for higher selectivity indexes (SI). Contrarily, some other conclusions of the previous work have now been clarified, for example, the presence of a 3′-substitution does not always promote an increase in SI by decreasing the derivative’s toxicity levels, or the introduction of a 3,6-diNCH_3_ pattern being crucial to the antileishmanial activity of the compounds (Figure 5).

**Figure 6:**
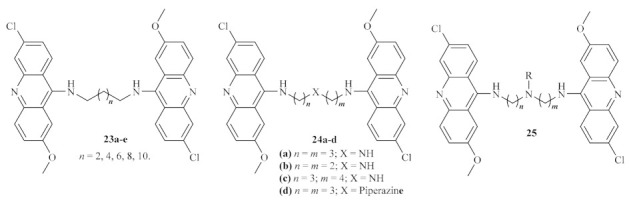
Bis (9-amino-6-chloro-2-methoxyacridine) derivatives evaluated against *L. infantum*.

At the beginning of the century, a research group synthesized a huge series of forty bis (9-amino-6-chloro-2-methoxyacridine) derivatives using several linkers between the two acridine fragments, such as alkanediamines, polyamines, or side chain-containing polyamines (Figure 6) [26]. These series of compounds were further evaluated for their activity against some different protozoan parasites, with particular interest in the amastigote stage *of L. infantum*. The variety of linkers evaluated allows a deeper understanding of the optimal type and length to be introduced between the two acridine fragments in order to enhance their antileishmanial properties. Curiously, except for the piperazine-containing derivative, the first two series of compounds **23** and **24** revealed high levels of cytotoxicity, which might be associated with strong interactions with DNA, particularly characteristic of bis-intercalation [27]. From the third series of compounds **25**, only five demonstrated the absence of cytotoxicity. These compounds are characterized by the presence of a polar group, such as an amine, alcohol, or carboxylic acid, in the side chain attached to the linker. Even though some of the compounds evaluated displayed high antileishmanial activity (half maximal inhibitory concentration (IC_50_) as low as 1.56 µM), the majority are not suitable as potential antileishmanial agents due to their high cytotoxicity. In turn, those with low or no levels of cytotoxicity were revealed to be only slightly effective against *L. infantum*, without considerable activity.

**Figure 7:**
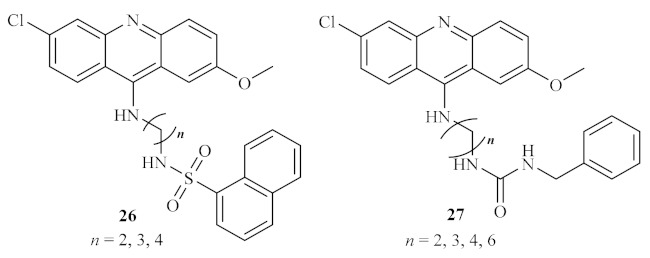
Sulfonamide **26** and urea **27** analogs of quinacrine against *L. donovani*.

A year later, based on the proven antiprotozoal properties of quinacrine **10** (Figure 3), some sulfonamide **26** and urea analogs **27** of quinacrine were synthesized and evaluated against a few types of protozoan, particularly against *L. donovani* amastigotes [28]. These derivatives are characterized by the presence of methylene groups with terminal sulfonamide and urea moieties between the acridine fragment and another hydrophobic scaffold (Figure 7). Therefore, the introduction of these groups is intended to improve the solubility of this type of compound. In addition to the most common in vitro assays against the parasite amastigote form, these compounds were also evaluated for their inhibitory activity against the trypanothione reductase enzyme (TR). TR is the enzyme responsible for maintaining trypanothione in its reduced state and, consequently, guaranteeing the proper function of the trypanothione pathway, a metabolic pathway essential for parasite growth and survival by regulation of oxidative stress [29]. Thus, this enzyme has been widely depicted as a potential molecular target for developing novel antiprotozoal agents. This research demonstrated that these derivatives could considerably inhibit the TR enzyme, with IC_50_ levels ranging from 3.3 µM to 19.3 µM. Furthermore, the results also show that these compounds display significant effects against the parasite by suppressing the survival and growth of the parasite, with median effective dose (ED_50_) values below 1.0 µM. From a metabolic perspective, considering that the compounds are more active against the parasite itself than against a specific enzyme (in this case, the TR enzyme), it is possible to suggest that these compounds might act against more than one metabolic pathway of the parasite. However, these compounds present high toxicity levels, which impairs their potential use to develop novel antileishmanial agents.

**Figure 8:**
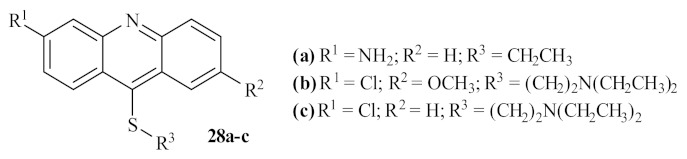
Acridine thioethers **28** against *L. donovani*.

In 2002, another French research group performed a thorough study of the antileishmanial potential of three acridine thioethers **28** (Figure 8) against *L. donovani*, both in vitro and in vivo [30]. The results demonstrated that the first two derivatives (**28a** and **28b**) are more effective against the promastigote forms of *L. donovani* than the third acridine-based compound evaluated **28c**, being able to considerably inhibit the parasite’s growth at the 0.39 µM (1 µg/mL), 0.27 µM (1 µg/mL), and 2.9 µM (0.1 µg/mL), respectively. In addition, considering the effect of these compounds on the in vitro growth of the amastigote forms of *L. donovani*, the derivative **28b** emerged as the most active derivative at the lowest concentration, leading to an inhibition of 43% at 0.27 µM (0.1 µg/mL). In contrast, the remaining derivatives appear to be virtually inactive against the amastigote form of this parasite. In turn, the in vivo studies demonstrated that these compounds could also decrease the percentage of parasitation in infected rats, with particular emphasis on derivative **28b**. In conclusion, these authors, in association with the results of previous studies developed on *Trypanosoma cruzi* [31], suggest that the antiparasitic activity of these acridine derivatives is closely related to the interaction between the DNA of the parasite and the compounds, particularly when the sulfur atoms are substituted with an alkylaminoalkyl group. Additionally, from a morphological perspective, these compounds can also promote a separation of the nuclear membrane from the chromatin, leading to the death of the parasite by apoptosis.

**Figure 9:**
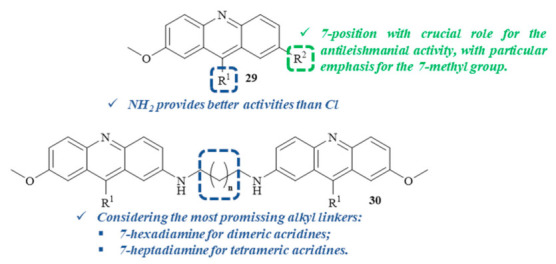
Most promising 7-substituted 9-(chloro or amino)-2-methoxyacridine derivatives against *L. infantum*.

A year later, two series of 7-substituted 9-(chloro or amino)-2-methoxyacridines **29**, as well as some corresponding unsubstituted dimers **30** and tetramers (Figure 9), were evaluated for their in vitro antiproliferative activity against both forms of *L. infantum*, with a focus on understanding their potential mechanisms of action [32]. Considering the toxicity levels of these series of compounds against human monocytes, it is important to mention that, with a few exceptions, most of the compounds from both series displayed low toxicity levels. In relation to their antileishmanial activity, the results demonstrated that the 9-aminoacridines are more active than the corresponding 9-chloroacridines, with the first group being more capable of affecting parasite growth and survival. Furthermore, the 7-substituent also plays a crucial role in the inhibitory activity of these compounds, with special emphasis on the introduction of a 7-methyl group, making 9-amino-2-methoxy-7-methylacridine and 9-chloro-2-methoxy-7-methylacridine the most promising antileishmanial compounds of the single acridine series. Thus, these monomers were used to obtain the corresponding dimeric and tetrameric derivatives, using several alkanediamines as linkers. In the case of the dimeric derivatives, the compound containing a 7-hexanediamine linker demonstrated the most promising selective antileishmanial activity. In contrast, the 7-heptanediamine linker was revealed to be the optimal linker for the antileishmanial activity of tetrameric derivatives. However, dimeric and tetrameric complexes presented lower activities than their corresponding monomers.

Regarding these compounds’ potential mechanism of action, this research work suggests that the antileishmanial properties demonstrated by these acridine derivatives might be closely related to a multitarget approach. This multitarget mechanism is primarily associated with inhibiting the DNA metabolism through DNA intercalation or even the inhibition of the processes involving DNA–protein interactions and, additionally, with effects on many other metabolic pathways, such as protein and lipid metabolisms [32].

**Figure 10:**
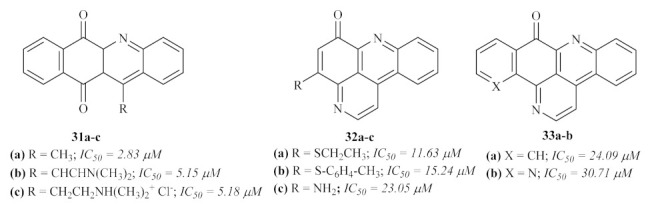
Marine pyridoacridone alkaloids against *Leishmania* promastigotes.

In the same year, a series of marine pyridoacridone alkaloids were evaluated for their in vitro antileishmanial activity against *L. donovani* promastigotes and amastigotes, including ascididemin and other related synthetic derivatives [33]. The results demonstrated that some of the evaluated derivatives present considerable antileishmanial activity, even if none of it reaches the values of the reference drug amphotericin B. Considering the activity against the promastigote stage, a series of eight compounds (**31**, **32**, and **33**, Figure 10) demonstrated significant levels of activity, with the remaining derivatives presenting moderate to no activity. However, due to its high cytotoxicity values against mammalian cells, none of these derivatives were further evaluated for their antileishmanial activity against the amastigote stage of the parasite.

**Figure 11:**
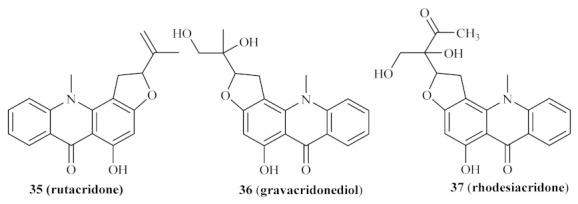
Acridones isolated from *Thamnosma rhodesica* (Bak. f.) Mendonça.

In 2004, Ahua et al. isolated several compounds from *Thamnosma rhodesica* (Bak. f.) Mendonça, a plant commonly used in Zimbabwe as an ant and flea repellent or for relieving chest pain, from which we must highlight four acridones [34]. These compounds were then evaluated against both promastigote and amastigote forms of *L. major*, with the results demonstrating that some of these derivatives present promising antileishmanial properties. From the four acridones evaluated, rutacridone **35**, gravacridonediol **36**, and rhodesiacridone **37** (Figure 11) demonstrated similar efficiencies against the promastigote form of the parasite, at 10 µM, by inhibiting its survival by 63.1%, 46.0%, and 69.3%, respectively. At the same concentration, none of these derivatives demonstrated considerable toxicity to mammalian macrophages, allowing its evaluation against the intracellular amastigotes. Interestingly, the effect of gravacridonediol **36** and rhodesiacridone **37** turned out to be even more pronounced against the amastigote form of *L. major* by inhibiting this form by 90.5% and 93.8%, respectively.

**Figure 12:**
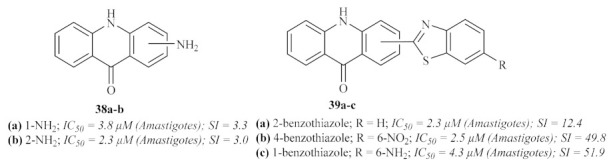
Acridone–benzothiazole hybrids against *Leishmania*.

In the same year, the Delmas group synthesized a series of acridine–benzothiazole hybrids, namely acridin-9-(*10H*)-ones substituted with amino or (1,3-benzothiazol-2-yl)-amino groups, and evaluated them for their in vitro antileishmanial properties against both stages of *L. infantum* (Figure 12) [35]. This research work demonstrated that the antileishmanial activity of this type of compound is highly dependent on the nature and position of the substituted benzothiazole fragment. Furthermore, the addition of a benzothiazole group is also responsible for decreasing the compounds’ cytotoxicity, which contributes to more suitable SI. Considering the type of benzothiazole introduced in the acridone scaffold, this series of compounds may be further divided into four smaller groups: the simple amino acridones and the unsubstituted benzothiazole, nitrobenzothiazole, and aminobenzothiazole.

The first group of compounds **38**, those without any benzothiazole group, demonstrated low to no antileishmanial activity against the promastigote stage of the parasite (IC_50_ values from 21 µM to >200 µM). Against the amastigotes, these compounds were considerably more effective, with the presence of a 1-NH_2_ **38a** or a 2-NH_2_ **38b** being the optimal positions for the antileishmanial activity of this group of compounds. However, these compounds also demonstrated high toxicity levels, resulting in weak SI values of 3.3 and 3.0, respectively.

As mentioned above, the introduction of the benzothiazole promoted better SI levels, regardless of its position, and enhanced efficiency against both promastigotes and amastigotes, leading to more promising antileishmanial agents. Considering the unsubstituted benzothiazole, except for the 4-position that displayed only moderate activity, the introduction of this group led to high levels of antileishmanial activity against both forms of the parasite. Amongst these derivatives, 2-(benzothiazol-2-ylamino)-10*H*-acridin-9-one **39a** emerged as the most promising antileishmanial agent, converging high activity and TI levels. In turn, introducing a 6-nitro group on the benzothiazole ring tends to decrease this class of compound’s efficiency against promastigotes, with IC_50_ values above 200 µM. However, 4-(6-nitrobenzothiazol-2-ylamino)-10*H*-acridin-9-one **39b** displayed high antileishmanial activity against *Leishmania* amastigotes associated with an elevated SI. Finally, with only one exception, the presence of a 6-amino group on the benzothiazole led to a decrease in the antileishmanial activity against promastigotes. The exception contemplates 1-(6-aminobenzothiazol-2-ylamino)-10*H*-acridin-9-one **39c**, a compound highly capable of inhibiting the amastigote form of the parasite and rendering an elevated SI value.

**Figure 13:**
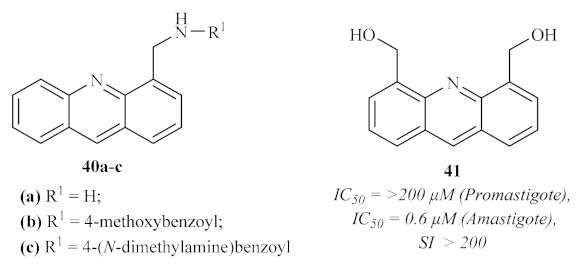
Most promising antileishmanial 4-mono and 4,5-disubstituted acridines.

One year later, the same research group continued focusing their efforts on evaluating acridine’s antileishmanial potential by synthesizing and evaluating the antileishmanial activities of both 4-mono and 4,5-disubstituted acridines (Figure 13) [36]. The results of this study demonstrated that the mono-substituted acridines present stronger antileishmanial activities than their disubstituted homologs, with the latter group being weakly active against the promastigote form of the parasite. Furthermore, it became clear that compounds containing a nitrogen bond are also more active than those with an oxygen bond. Notably, in the case of the mono-substituted acridines, only one compound demonstrated considerable inhibitory activity against the promastigote form of the parasite, consisting of the 4-ethylaminoacridine **40a** (IC_50_ = 1.7 µM; TI = 2.9), while two displayed promising selective anti-amastigote activities, characterized by a 4-methoxybenzoyl **40b** (IC_50_ = 9.4 µM; TI = 42.8) and 4-(*N*-dimethylamino)benzoyl **40c** (IC_50_ = 4.7 µM; TI = 30.4) groups, respectively. Regarding the 4,5-disubstituted acridines, some of these derivatives demonstrated considerable antileishmanial activity against both forms of the parasite with emphasis on 4,5-bis (hydroxymethyl)acridine **41** (IC_50_ = 0.6 µM; TI > 200). Interestingly, several disubstituted acridines were characterized by an amastigote-specific activity, which might suggest that these compounds may act against the *Leishmania* parasite through a different mechanism of action, unrelated to DNA intercalation. This assumption is supported by the fact that these acridine derivatives are capable of preventing macrophage infection through the reduction of parasite internalization, in addition to their selective antileishmanial activity against amastigotes.

**Figure 14:**
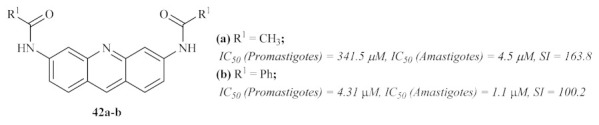
Structures of *N*-[6-(acetylamino)acridin-3-yl]acetamide **42a** and *N*-[6-(benzoylamino)acridin-3-yl]benzamide **42b**.

In 2007, Di Giorgio et al. synthesized two more series of acridine derivatives, monofunctional 6-substituted acridine and bifunctional 3,6-disubstituted acridine derivatives, and evaluated their antileishmanial activities [37]. The results of this research work demonstrated that some derivatives from this particular type of acridines present promising antileishmanial activities against both forms of the parasite. However, due to the high cytotoxicity shown by many of the derivatives, it becomes crucial to emphasize *N*-[6-(acetylamino)acridin-3-yl]acetamide **42a** and *N*-[6-(benzoylamino)acridin-3-yl]benzamide **42b**, which display the highest levels of selectivity (Figure 14).

This study also allowed us to identify some molecular features that promote the specificity of the compounds for *Leishmania* parasites instead of human cells, with variations depending on the parasite’s form. Against the promastigote form, the optimal substituent pattern consists of two benzoylamino groups in disubstituted derivatives. The introduction of 4′-chloro or 4′-fluoro atoms leads to a significant increase in antileishmanial activity. In turn, against the amastigote form of the parasite, both 3,6-acetylamino and 3,6-benzoylamino groups are the best features for this activity, suggesting the necessity for a symmetric conformation for a specific anti-amastigote activity. From a mechanism of action perspective, it became clear that, despite the proven ability of acridines to bind DNA [38,39,40], their antileishmanial activity is not related to DNA binding. Instead, this might be associated with interferences on *Leishmania*–macrophage interactions or even the interaction of acridine derivatives with several cellular structures.

**Figure 15:**
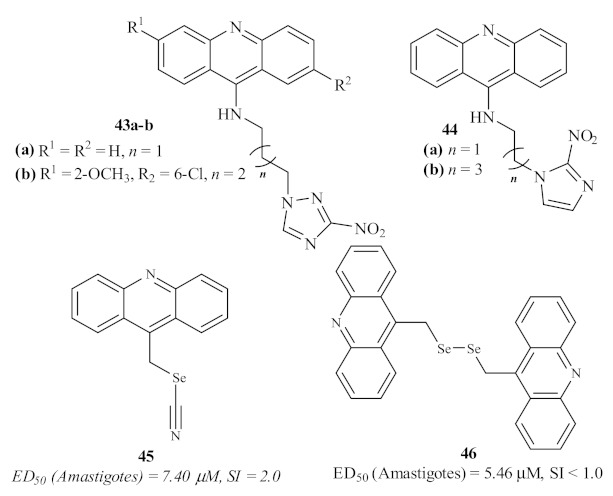
Antileishmanial acridine derivatives with triazole, imidazole, and selenium features.

Based on the current use of benznidazole for the treatment of Chagas disease, Papadopoulou et al. screened a series of forty-two 3-nitro-1*H*-1,2,4-triazole-based and 2-nitroimidazole-based aromatic and aliphatic amines for their antiparasitic properties, with emphasis on 4 acridine derivatives (Figure 15) [41]. Even though these derivatives demonstrated considerable levels of antileishmanial activity against axenic amastigotes of *L. donovani*, with IC_50_ values from 9.09 to 35.37 µM, their cytotoxicity levels were even higher, with IC_50_ values from 0.308 to 20.52 µM.

In 2016, Baquedano et al. evaluated a series of twenty-three heteroaryl selenocyanates and diselenides for their antileishmanial activity against the amastigote form of *L. infantum*, including two acridines (Figure 15) [42]. Through the incorporation of these two functionalities bearing selenium atoms, this group intended to perform a rational design of potent and selective antileishmanial agents. These acridines, acridin-9-ylmethyl selenocyanate **45** and 9,9-(diselenodiyldimethanediyl)diacridine **46**, demonstrated good levels of antileishmanial activity, with the diselenide-containing derivative being more active than the one containing the selenocyanate moiety. However, due to their high levels of cytotoxicity, these acridine derivatives were revealed not to be suitable for further development of novel antileishmanial agents.

**Figure 16:**
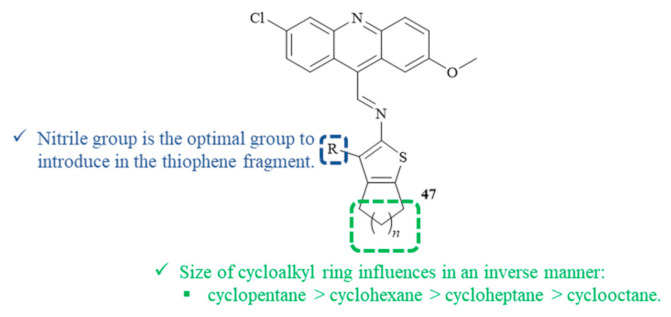
Thiophene–acridine hybrids **47** as antileishmanial agents.

In 2018, a Brazilian research group synthesized and evaluated eight compounds containing both 2-aminocycloalkyl[*b*]thiophene and acridine fragments for their antileishmanial activity against the promastigote form of *L. amazonensis* (Figure 16) [43]. Concerning the cytotoxicity evaluation, it is essential to mention that none of the thiophene–acridine hybrids showed any cytotoxicity against human erythrocytes up to 1000 µM. Regarding their antileishmanial activity, the hybrids containing the 6,9-dichloro-2-methoxyacridine and the 2-aminothiophene fragments **47** demonstrated good antipromastigote activity with IC_50_ levels from 9.60 µM to 69.11 µM. Structurally, the size of the cycloalkyl ring of the thiophene fragment clearly influences the compound’s antileishmanial activity through an inverse manner, following the order cyclopentane > cyclohexane > cycloheptane > cyclooctane. Furthermore, the substituent group present in the thiophene fragment also influences the antileishmanial activity, emphasizing the introduction of a nitrile radical. Based on these shreds of evidence, the most effective antileishmanial agent derived from this research work consist of the hybrid containing the nitrile group and a cyclopentane simultaneously, with an IC_50_ of 9.60 µM. Through some molecular docking studies, this research group suggests that the antileishmanial activity might be related to the potential inhibition of the enzyme pyruvate kinase, which, in association with the ability of the acridine fragment to intercalate with DNA, indicates a dual mechanism of action.

**Figure 17:**
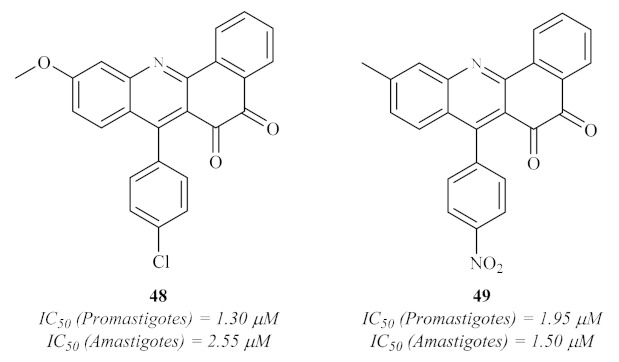
Antileishmanial 7-arylbenzo[*c*]acridine-5,6-diones against *L. donovani*.

In the same year, following the previous works from Giorgi et al. [37], Mahajan et al. designed, synthesized, and evaluated a series of thirty-three 7-arylbenzo[*c*]acridine-5,6-diones (Figure 17) for their potential against both promastigote and amastigote form of *L. donovani* [44]. The results demonstrated that several derivatives present significant antileishmanial activity against both promastigotes and axenic amastigotes. Interesting to mention is the fact that, against promastigotes, three compounds demonstrated activity levels similar to pentamidine (IC_50_ = 1.89 µM); six compounds showed considerable antileishmanial properties (IC_50_ from 2.00 µM to 2.56 µM); and ten were moderately active (IC_50_ from 4.03 µM to 10.44 µM). In turn, against amastigotes, four derivatives were clearly more active than pentamidine (IC_50_ = 9.57 µM), with IC_50_ values of 1.50–1.95 µM, and fifteen others were demonstrating significant activities, with IC_50_ values below 11.31 µM. In the case of the antipromastigote activity, the optimal substitution pattern consists of combining a 9-(4-chlorophenyl) group and a 2-methoxy group **48**, while the optimal anti-amastigote activity is composed of a 9-(4-nitrophenyl) group and a 2-methyl group **49**.

**Figure 18:**
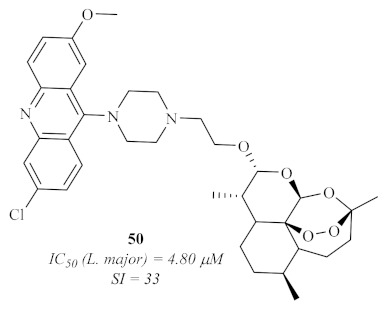
Artemisinin–acridine hybrid **50** with antileishmanial activity.

In 2020, based on previous evidence of the antiparasitic properties of artemisinin analogs [45], Aucamp et al. synthesized a series of acridines and artemisinin–acridine hybrids (Figure 18). They evaluated them for their in vitro antileishmanial activity against the promastigote form of three different strains of *Leishmania*, two of *L. donovani* and one of *L. major* [46]. The results of this research demonstrated that all the acridine derivatives presented significant levels of activity against all the *Leishmania* strains, with IC_50_ values in the micromolar range. However, with one exception, these derivatives also showed high levels of cytotoxicity, leading to undesirable SI (<9), which hinders the possibility of these compounds being used to develop novel antileishmanial agents. The exception consisted of a particular artemisinin–acridine hybrid **50** (Figure 18) that demonstrated a promising SI (33), with low levels of cytotoxicity (>100 µM) and a considerable level of antileishmanial activity, especially against *L. major* (4.80 µM). Important to mention is the fact that the hybridization of the acridine derivatives with artemisinin promotes an increase in the antileishmanial activity while decreasing the cytotoxicity levels of the derivatives, suggesting that acridine hybrids with other pharmacophores might be a solution to overcome acridine’s toxicity.

## 3. Xanthenes

**Figure 19:**
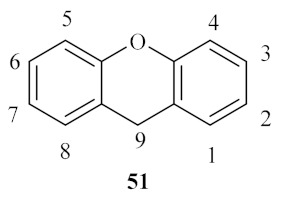
Xanthene basic scaffold and correspondent numbering system.

The class of xanthene derivatives **51** constitutes an important class of oxygen-containing tricyclic compounds, characterized by a dibenzo[*b,e*]pyran core, widely associated with a broad range of pharmaceutical and biological properties, such as antimicrobial, antiproliferative, antiviral, antioxidant, and anti-inflammatory activities, amongst others (Figure 19) [47,48].

Even though numerous reports have described these compounds as constituents of natural extracts throughout the past decades, the majority of the known xanthenes are synthesized. The synthesis of this type of compound might be performed through the cyclization of specific building blocks or by modifying a most commonly found class of xanthene derivatives, namely xanthones. This versatile structural fragment allows the introduction of virtually any substituent group in its core. Depending on the substituent group desired, different synthetic approaches might be performed to obtain the intended xanthene derivative. Sen et al. described one of the first syntheses of the xanthene nucleus in 1925, in which 3-hydroxyxanthene was obtained through the condensation of saligenin and resorcinol [49]. After that, numerous synthetic methods for the obtention of xanthenes with distinct substitution patterns have been described in literature, including the functionalization of particular core positions to synthesize broad spectra of xanthenes and the application of greener procedures [50,51,52]. Thus, this has allowed the use of xanthene in a huge range of biological activities, leading to the increasing importance of xanthenes in medicinal chemistry [48]. Besides its biological properties, this type of compound has been widely used for a broad range of applications, such as sensitizers in PDT, dyes in the food industry, industrial materials, and chemical probes for the visualization of biomolecules [52,53,54,55].

**Figure 20:**
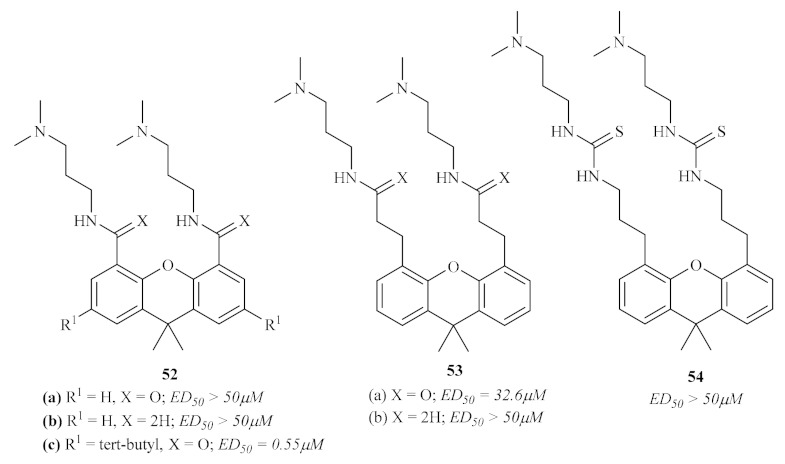
9,9-Dimethylxanthene tricyclics against TR and *L. donovani*.

By the end of the 20th century, through bibliographic research, Chibale et al. understood that several aromatic hydrophobic tricyclic moieties had already been reported as competitive trypanothione reductase (TR) inhibitors [56]. Thus, based on this knowledge, the research group decided to synthesize and evaluate a novel series of 9,9-dimethylxanthene tricyclics (**52**, **53**, and **54**) against *L. donovani* through the assessment of their trypanothione reductase (TR) inhibitory potential (Figure 20). Their results demonstrated that, considering the inhibition of TR, compounds containing longer carbon chains (two **53** or three methylene groups **54**) between the tricyclic moiety and the secondary amino group show stronger inhibition of TR in comparison with those containing only one **52b** or no methylene spacer **52a**. Interestingly, the efficiency of these compounds against TR was not replicated for the in vitro studies against the amastigote stage of *L. donovani*, with compound **52c** being the most effective (ED_50_ = 0.55 µM), suggesting that TR is not the target of its activity. This lack of correlation between TR inhibition and in vitro sensitivity of *L. donovani* amastigotes may be explained by the fact that TR inhibition is not entirely responsible for the effect of these compounds on the amastigotes. In turn, cell penetration and the parasite’s metabolism might also play a crucial role in the efficiency of these compounds against amastigotes.

**Figure 21:**
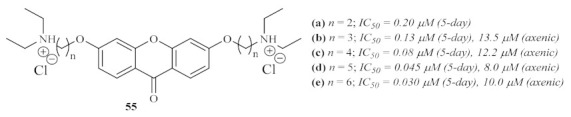
General structure of the 3,6-bis-ω-diethylaminoalkoxyxanthones **55**.

Three years later, in 2003, another research group focused their efforts on using a particular series of heme-binding xanthones, 6-bis-ω-diethylaminoalkoxyxanthones **55**, against *L. mexicana* (Figure 21) [57]. These compounds were selected since xanthones have already been described as potential antimalarial agents by both forming stable soluble complexes with heme and preventing their aggregation into insoluble hemozoin. Furthermore, it has been observed that these dibasic compounds accumulate in the parasite’s acidic vacuole and, considering the peculiar nutritional necessities of *Leishmania*, it was expected that the parasites would be significantly sensitive to the effects of heme-binding xanthones. The results of this research work revealed that all the evaluated derivatives present strong effects against both intracellular amastigotes and axenic amastigotes of *L. mexicana*, with IC_50_ values in the nanomolar range. Regarding the 5-day assay, it was possible to verify the strong influence of chain length on the compounds’ antileishmanial activity, with the activity increasing with the number of methylene groups in the carbon chain. Another piece of evidence obtained through this work contemplates a strong correlation between the heme affinity of the compounds and their antileishmanial activity. Interestingly, despite the same order of efficiency, these compounds were considerably less effective against the axenic amastigotes. Finally, considering all the evidence, this group proposes that these compounds undergo a cationization and accumulation process within the acidic phagolysosomes of the host, being further capable of effectively complexing with any source of heme and, consequently, starving the intracellular parasites.

**Figure 22:**
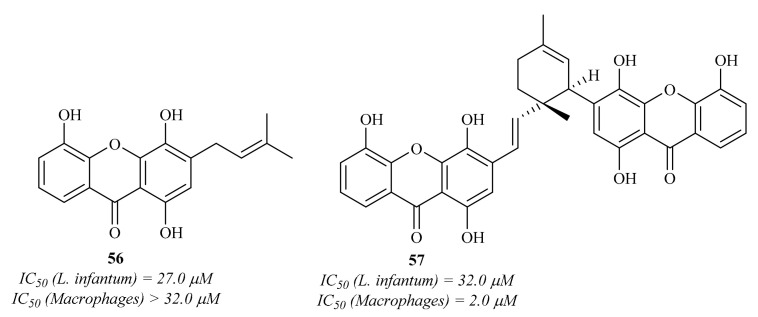
1,4,5-Trihydroxy-3-(3-methylbut-2-enyl)-9*H*-xanthen-9-one **56** and garcilivin A **57**, natural xanthones isolated from the root bark of *Garcinia livingstonei* T. Anderson.

Two years later, Mbwambo et al. isolated from the root bark of *Garcinia livingstonei* T. Anderson and evaluated a series of xanthones for their antiparasitic activities against the intracellular amastigotes of *L. infantum* [58]. The results of this work demonstrated that, from the five isolated xanthones, only two present considerable antileishmanial activity (Figure 22), emphasizing 1,4,5-trihydroxy-3-(3-methylbut-2-enyl)-9*H*-xanthen-9-one **56**. However, these compounds also demonstrated high cytotoxicity values, with compound **57** being even more effective against macrophages than against the *Leishmania* parasites.

**Figure 23:**
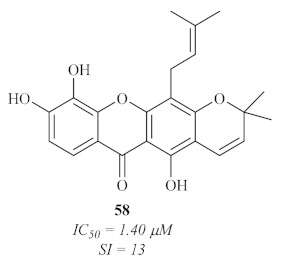
Xanthone V_1_ **58**, promising antileishmanial agent isolated from *S. globulifera* leaves.

In 2007, an international research group decided to study the phenolic compounds from two Cameroonian medicinal plants, *Allanblackia monticola*, and *Symphonia globulifera* [59]. Amongst the entire phenolic content of these plants, three xanthones were isolated and evaluated against the axenic amastigotes of *L. donovani*, with only one of them **58** (Figure 23) presenting both promising antileishmanial activity and cytotoxicity levels.

**Figure 24:**
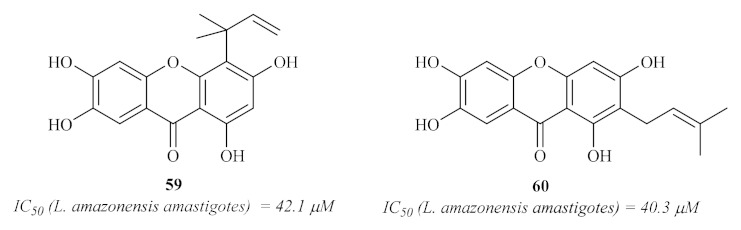
Xanthones with antileishmanial activity isolated from *Allanblackia gabonensis*.

In 2008, Azebaze et al. isolated seven xanthones from the stem bark of *Allanblackia gabonensis* and evaluated them against axenic amastigotes of *L. amazonensis* [60]. These preliminary studies showed that some of these xanthones present promising antileishmanial activities, emphasizing the tetraoxygenated prenylated xanthones activities when compared with the ones presented by dioxygenated simple xanthones. Additionally, the results suggest that their antileishmanial activity might be related to the presence of the 2- or 4-prenyl group. From the seven isolated xanthones, it is important to emphasize compounds **59** and **60** (Figure 24) as the most effective against the *L. amazonensis* axenic amastigotes.

**Figure 25:**
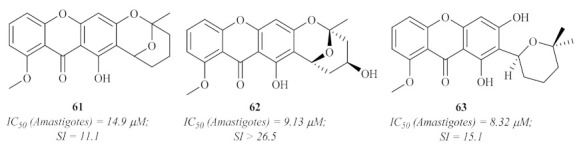
Xanthones with antileishmanial activity isolated from the marine-derived fungus *Chaetomium* sp.

This same year, another research group focused on characterizing the natural compounds from the marine-derived fungus *Chaetomium* species. This led to the isolation of three new xanthones with unusual and rare structural features (Figure 25) [61]. Thus, these novel xanthone derivatives were evaluated for their antileishmanial potential against the amastigotes of *L. donovani*. The results demonstrated that all the evaluated xanthones present considerable antileishmanial potential in the micromolar range.

**Figure 26:**
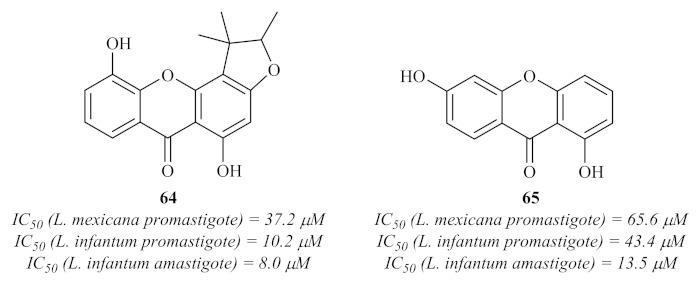
Xanthones with antileishmanial activity isolated from *Garcinia vieillardii* Pierre.

Nevertheless, in 2008, Hay et al. evaluated the polyphenolic content of an endemic plant from New Caledonia, *Garcinia vieillardii* Pierre, against both *L. mexicana* and *L. infantum* [62]. Five xanthones were further isolated from this phenolic content, characterized and evaluated for their antileishmanial properties, with only two of them (**64** and **65**) showing considerable activity against the promastigote stage of both species of *Leishmania* (Figure 26). Regarding their potential against the amastigote stage of the parasite, these two compounds also presented significant activity levels, demonstrating that these two acridones are efficient against both stages of the parasite and capable of passing through the macrophage membranes.

**Figure 27:**
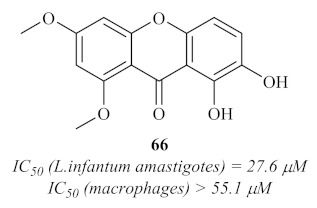
Antileishmanial xanthone isolated from the roots of *Andrographis paniculate* (Burm.f.) Nees.

The search for antileishmanial agents from natural sources continued throughout the decade, and a year later, in 2009, another research group isolated four xanthone derivatives from the roots of *Andrographis paniculate* (Burm.f.) Nees [63]. These xanthones were evaluated in vitro for their antiprotozoal activity, particularly against the amastigotes of *L. infantum*, and the results demonstrated that, from the four isolated xanthones, only one (**66**) showed considerable antileishmanial activity, with an IC_50_ value of 27.6 µM (Figure 27).

**Figure 28:**
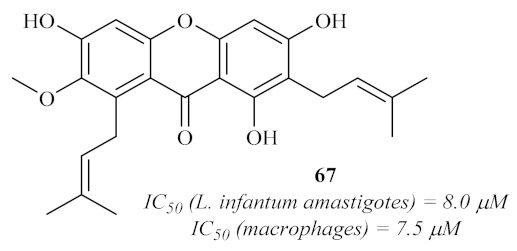
α-Mangostin **67**, major constituent of *Garcinia mangostana* Linn.

A few years later, in 2013, another research group focused their efforts on evaluating the in vitro antileishmanial of both dichloromethane and ethyl acetate extracts of *Garcinia mangostana* Linn. against *L. infantum* amastigotes [64]. Furthermore, this antileishmanial evaluation was also extended to the major constituent of the plant, namely α-mangostin **67** (Figure 28). Considering the evaluation of this particular compound, the results demonstrated a promising antileishmanial potential, with an IC_50_ of 8.0 µM. However, its cytotoxicity is even higher (IC_50_ = 7.5 µM), which disqualifies its potential use against leishmaniasis. Interesting to verify is the fact that, as in the works of Azebaze et al. [60], multiple hydroxy groups and a 2-prenyl group seem to be a key feature for the high antileishmanial activity of this type of compound.

**Figure 29:**
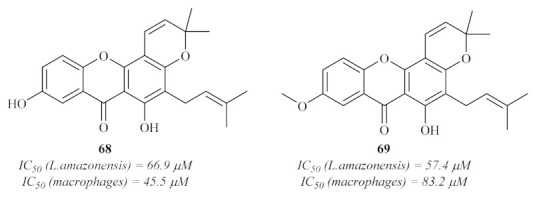
Prenylated xanthones isolated from *Clusia pernambucensis* G. Mariz.

In the same year, a Brazilian research group evaluated the antileishmanial potential of two xanthones from *Clusia pernambucensis* G. Mariz. against *L. amazonensis* amastigotes (Figure 29) [65]. Based on the results, these two xanthones only demonstrated moderate activity against *L. amazonensis*, being still the most active compounds isolated from this plant. Thus, these results corroborate the works of Azebaze et al. [60], Dua et al. [63], and Kelly et al. [57], in which both the importance of prenyl groups at 2- and 4-positions, as well as the hydroxy and methoxy groups, were previously described against other species of *Leishmania* (*L. infantum* and *L. mexicana*). Despite their antileishmanial potential, these compounds also demonstrated high levels of cytotoxicity against murine macrophages, with IC_50_ values similar to those observed against the parasite.

**Figure 30:**
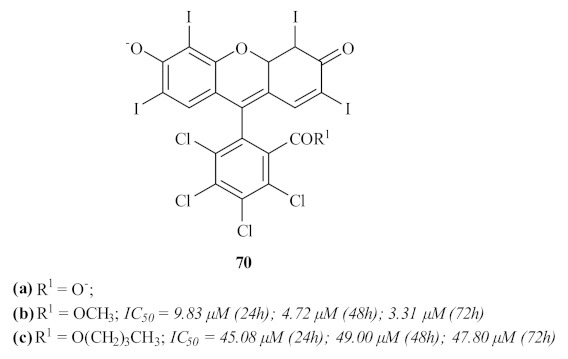
Xanthone-type compounds for application in photodynamic therapy.

A few years later, another Brazilian research group intended to apply the widely studied technique of photodynamic therapy (PDT) to treat cutaneous leishmaniasis through the use of three promising xanthones [66]. The selective derivatives, namely xanthene rose bengal B **70a** and its correspondent methyl **70b** and butyl esters **70c**, presented favorable characteristics for their use in PTD, and their antileishmanial activity was assessed against *L. amazonensis* promastigotes (Figure 30). Through their use in PDT, the results showed that only the xanthene rose bengal B **70a** did not present any activity against the promastigotes. In turn, the other two compounds (**70b** and **70c**) demonstrated considerable activity values in an illumination-dependent setting, with compound **70c** as the most active of the two. Despite their proven antileishmanial potential, these compounds demonstrated high levels of cytotoxicity against macrophages, which not only makes their evaluation against the intracellular amastigotes of the parasite impossible but also impairs any further use of these compounds for the generalized treatment of leishmaniasis. Nevertheless, the authors do not rule out their potential application as photosensitizers in PDT to treat cutaneous leishmaniasis, the appropriate evaluation of their absorption and toxicity being crucial for this purpose.

**Figure 31:**
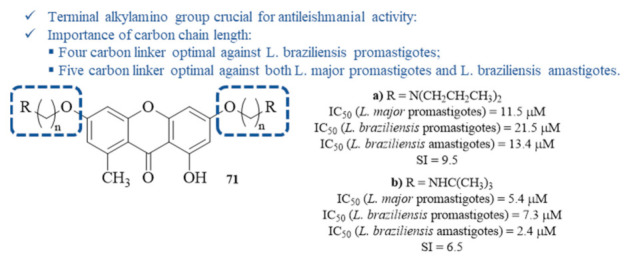
Structure–activity relationship with most effective antileishmanial xanthones from the series of ω-aminoalkoxylxanthones **71**.

By the beginning of this decade, Micheletti et al. synthesized and evaluated a series of xanthones containing a diverse range of side chains at 3- and 6-positions for their antileishmanial activity against *L. major* promastigotes and *L. braziliensis* promastigotes and amastigotes (Figure 31) [67]. Considering the antileishmanial activity against the parasite’s promastigote stage, most of the derivatives containing terminal alkylamino groups demonstrated higher activity levels than the positive control, amphotericin B, being more active against *L. braziliensis*. Thus, the most active compound against *L. braziliensis* contains a four-carbon spacer between the amino group and the oxygen atom, with an IC_50_ value of 0.1 µM, while the most active compound against *L. major* bears a five-carbon spacer, with an IC_50_ value of 5.4 µM. Against the *L. braziliensis* amastigotes, the most active is also the compound bearing a five-carbon linker, with an IC_50_ value of 2.4 µM. Furthermore, the wide variety of substituent groups enables an interpretation of the result to establish a structure–activity relationship study. This study, corroborating another work conducted against *L. Mexicana* [57], demonstrated that, in general, the xanthones bearing terminal alkylamino groups are highly effective against *Leishmania*, with a clear correlation with the length of the carbon chain. The remaining compounds, lacking a side carbon chain with a terminal alkylamino group, only showed moderate to no activity against both the promastigotes and amastigotes of *Leishmania*, confirming the relevance of this particular structural feature to the antileishmanial activity. Finally, all these compounds were evaluated for their cytotoxicity, demonstrating that some also present high activity levels against VERO cells. Determining the SI makes it important to highlight compounds **71a** and **71b** as the safest to use against Leishmania, with compound **71a** presenting the highest SI despite its equally high IC_50_ of 13.4 µM against *L. braziliensis* amastigotes.

## 4. Conclusions and Future Perspectives

As of now, there is no evidence for the development of an effective vaccine against *Leishmania*, with the use of organoantimonial compounds remaining as the first line of treatment against all forms of leishmaniasis. Throughout the years, the scientific community’s efforts have allowed the improvement of the currently used pentavalent antimonials and the development of novel drugs, such as amphotericin B, miltefosine, paromomycin, sitamaquine, and pentamidine, leading to more effective treatment with progressively fewer side effects of medicines. However, considering the complexity of the disease, the failure of the currently used pharmaceuticals, and the development of resistance mechanisms by the parasite, the objective of the WHO to eradicate leishmaniasis remains a virtually impossible endeavor.

In an attempt to overcome this huge obstacle, many families of compounds have been assessed for their potential as antiprotozoal agents, and more particularly, as antileishmanial agents [16,68]. However, the knowledge about acridine and xanthene derivatives as antileishmanial agents, as well as the optimal structural features to promote an efficient action against *Leishmania*, remain scarce in current literature. Thus, the information compiled in this review proves the potential of acridine- and xanthene-type compounds for the development of novel antileishmanial treatments, with some derivatives demonstrating high levels of efficiency in association with low values of cytotoxicity. Additionally, some structural functionalizations that promote the improvement of these compounds against *Leishmania* have also been depicted and are crucial to emphasize.

Considering the acridine-type compounds, some structural features emerged as crucial for the antileishmanial potential of these derivatives. In particular, the presence of a carbon chain linker and a terminal alkylamino group, independent of the position in which these are introduced, seems to be one of the main features to improve the acridine derivatives’ antileishmanial activity. Furthermore, the length of the carbon chain linkers proves to highly influence these properties in a direct association, with longer chains promoting better activities. This effect may also be related to an increase in the compounds’ lipophilicity. Even though the position of these features is not crucial, it became clear that the introduction of alkylamino groups at 3- and 6-positions may be the most advantageous for this type of property. Finally, throughout the literature, several examples of both dimers of a single pharmacophore and the hybridization of two distinct pharmacophores have been depicted as potential antileishmanial agents. However, while dimerization may not be the most suitable approach, mainly due to the loss of activity compared to the monomers, the hybridization of different pharmacophores might allow for overcoming one of the major drawbacks of using acridine derivatives, namely, their toxicity. Regarding the xanthene-type compounds, the structural features crucial for their antileishmanial activity are very similar to those already described for acridine derivatives, suggesting that this might be a particularity of this type of tricyclic compound. In addition to these structural characteristics, the presence of polyhydroxy substituents associated with 2- and 4-prenyl groups was revealed as a quite relevant substitution pattern for the antileishmanial activity of xanthenes.

In conclusion, this paper clearly demonstrates the potential of acridine and xanthene derivatives for discovering and developing a novel treatment for leishmaniasis. It provides the scientific community with a more in-depth insight into the optimal functionalization to perform in these compounds. However, some limitations for developing novel treatments against leishmaniasis must be mentioned. The reduced number of research works for the development of novel antileishmanial agents, in association with the high complexity of this disease, constitutes a major limitation for the arrival of new treatments against *Leishmania*, since the obtention of a single molecule capable of killing the parasite and overcoming its defense mechanisms, is a virtually impossible endeavor. Furthermore, the lack of enzymatic assays capable of assessing the metabolic pathways in which the synthesized molecules act to cause their antileishmanial effects constitutes another limitation, as it does not allow the confirmation of molecular targets affected by the molecules.

## Data Availability

Data sharing not applicable.
